# *Paenibacillus lentimorbus* Inoculation Enhances Tobacco Growth and Extenuates the Virulence of *Cucumber mosaic virus*

**DOI:** 10.1371/journal.pone.0149980

**Published:** 2016-03-02

**Authors:** Susheel Kumar, Puneet Singh Chauhan, Lalit Agrawal, Rashmi Raj, Ashish Srivastava, Swati Gupta, Shashank Kumar Mishra, Sumit Yadav, Poonam C. Singh, Shri Krishna Raj, Chandra Shekhar Nautiyal

**Affiliations:** 1 Plant Molecular Virology Laboratory, Council of Scientific and Industrial (CSIR)-National Botanical Research Institute (NBRI), Rana Pratap Marg, Lucknow (UP), India; 2 Division of Plant Microbe Interaction, CSIR-NBRI, Rana Pratap Marg, Lucknow (UP), India; National Institute of Plant Genome Research, INDIA

## Abstract

Previous studies with *Paenibacillus lentimorbus* B-30488” (hereafter referred as B-30488), a plant growth promoting rhizobacteria (PGPR) isolated from cow’s milk, revealed its capabilities to improve plant quality under normal and stress conditions. Present study investigates its potential as a biocontrol agent against an economically important virus, *Cucumber mosaic virus* (CMV), in *Nicotiana tabacum* cv. White Burley plants and delineates the physical, biophysical, biochemical and molecular perturbations due to the trilateral interactions of PGPR-host-CMV. Soil inoculation of B-30488 enhanced the plant vigor while significantly decreased the virulence and virus RNA accumulation by ~12 fold (91%) in systemic leaves of CMV infected tobacco plants as compared to the control ones. Histology of these leaves revealed the improved tissue’s health and least aging signs in B-30488 inoculated tobacco plants, with or without CMV infection, and showed lesser intercellular spaces between collenchyma cells, reduced amount of xyloglucans and pectins in connecting primary cells, and higher polyphenol accumulation in hypodermis layer extending to collenchyma cells. B-30488 inoculation has favorably maneuvered the essential biophysical (ion leakage and photosynthetic efficiency) and biochemical (sugar, proline, chlorophyll, malondialdehyde, acid phosphatase and alkaline phosphatase) attributes of tobacco plants to positively regulate and release the virus stress. Moreover, activities of defense related enzymes (ascorbate peroxidase, guaiacol peroxidase, superoxide dismutase and catalase) induced due to CMV-infection were ameliorated with inoculation of B-30488, suggesting systemic induced resistance mediated protection against CMV in tobacco. The quantitative RT-PCR analyses of the genes related to normal plant development, stress and pathogenesis also corroborate well with the biochemical data and revealed the regulation (either up or down) of these genes in favor of plant to combat the CMV mediated stress. These improvements led tobacco plant to produce more flowers and seeds with no negative impact on plant health. The present study may advocate the applicability of B-30488 for crop yield improvement in virus infested areas.

## Introduction

Plant growth-promoting rhizobacteria (PGPR), the advantageous colonized bacterial population associated with plant’s root, have been well documented for several beneficial attributes in crop plants. One of the earliest virtues associated with PGPR is the promotion of plant’s growth for high yield or biomass [1–3]. Another lucrative property of PGPR is to elicit the defense system in plants and to improve the plant’s ability to withstand abiotic stress conditions such as dry or cold weather, sodic or chemical amended soil etc. [4–6]. The applicability of PGPR as a biological control agent (BCA) for fungi [7,8], bacteria [9], and nematode [10] have been evaluated. And now, the implementation of PGPR as BCA is increasingly being accepted and has shown promising results in managing a wide spectrum of plant viruses in several crops or model plants under greenhouse as well as in field conditions [11–18]. However, literature revealed the inadequate studies to understand the further consequences of this trilateral interaction of PGPR-Host-Virus and no studies have been reported on structural (anatomical), biophysical and biochemical alterations that may have occurred due to PGPR and virus interaction. Here, attempts have been made to delineate the affects of a PGPR in *Nicotiana tabacum* cultivar White Burley plants (a well known model and commercially important plant) using an economically important plant virus, *Cucumber mosaic virus* (CMV) [19].

CMV is a member of genus *Cucumovirus* (family *Bromoviridae*) and is one of the most widespread plant viruses capable of infecting more than 1200 plant species including cereals, fruits, vegetables, ornamentals and other economically important plants [19]. The virus is readily transmitted in a non-persistent manner by more than 75 aphid species, therefore, limits agriculture worldwide [19]. Strains of CMV have been classified into two major subgroups: I and II, of which subgroup I has further been divided into two subgroups, IA and IB. In India, subgroup IB strains persist in high frequency (as compared to IA and II) and infect several crop plants [20–22], hence warrant a facile yet effective management strategy.

Earlier studies indicate that PGPR act as catalyst of induced systemic resistance (ISR) in plants [23]. Although, use of PGPR for management of viral diseases under field condition is a lesser practiced trend and only a few of studies exist for control of *Tomato mottle virus* [12] and *Tomato spotted wilt virus* [15] in tomato; CMV in tomato [13,24] and cucumber [24]; *Banana bunchy top virus* in banana [16,17]. These studies were related to the application of PGPR for control of virus through ISR and for the growth characters of the plants (e.g. increase in biomass, surface area of leaf, and root-shoot length); however, there exists a gap in overall understanding of the biophysical, biochemical, molecular and anatomical changes during PGPR-Host-Virus interaction. Here, we have evaluated the ISR elicitation by PGPR, *Paenibacillus lentimorbus* B-30488 [25], in tobacco plants against a pathogenic strain of CMV [26] prevalent in India.

## Materials and Methods

### PGPR strain and inoculum preparation

*P*. *lentimorbus* NRLL B-30488 (referred as B-30488) isolated from *Sahiwal* cow's milk [25] was used for this study. For inoculum preparation, B-30488 was cultured in nutrient broth (Himedia Pvt. Ltd., India) for 48 h at 30°C in an incubator shaker (Innova Model 4230, New Brunswick Scientific, Edison, NJ, USA) rotating at 200 rpm and used for soil inoculation.

### Virus (CMV) culture

#### Maintenance of CMV culture

A characterized strain of CMV-subgroup IB [26] was used throughout the study. CMV was maintained by sap inoculations [27] on *N*. *tabacum* cv. White Burley plants which were kept in an insect-proof glasshouse with ambient temperature of 25±2°C during day and 21±2°C in night regimes under natural illumination. All experiments were conducted in glasshouse at CSIR-National Botanical Research Institute, Lucknow, India (Lat. 26° 51' N; Lon. 80° 55' E).

#### Plant material and CMV inoculations

Tobacco cv. White Burley seeds were surface sterilized in 1% Ca(OCl)_2_ for 3 min, then rinsed several times with distilled water and were sown in seedling trays filled with autoclaved garden soil, and were grown to 3–4 leaves stage in glasshouse. To minimize the influence of an individual plant on the results three biological replicates of each treatment with 10 plants in each replicate were used. A set of 10 plants mock inoculated with virus inoculation buffer was included in experiments as control.

The virus inoculum for sap transmission assay was prepared using the juvenile tobacco leaf exhibiting severe mosaic symptoms due to CMV. Briefly, 1 g of leaf tissue was macerated in 10 ml of 50 mM potassium phosphate buffer (pH 7.0) supplemented with 10 mM sodium sulfite on ice. The suspension was squeezed through two layers of muslin cloth and sap obtained was mechanically rubbed on carborundum pre-dusted leaf of 3–4 leaf stage (four weeks old) tobacco seedlings. Severity of the disease symptom (mosaic, leaf deformation and stunting) was observed regularly till 28 days post inoculation (dpi).

### Experimental set up for virus challenge and B-30488 treatment

3–4 leaf stage tobacco seedlings were transplanted individually into 48 pots (with the same soil on which tobacco seeds were germinated and grown), and allowed for one week acclimatization to glasshouse conditions before experiment. These seedlings were subjected to the following four treatments with 12 pots each: pot soil drenched with nutrient broth served as control (designated as Control); soil drenched with B-30488 (B-30488); plant inoculated with virus (CMV); soil drenched with B-30488 following the CMV inoculation (B-30488+CMV). The 20 ml B-30488 cell suspension (9.0 log_10_ CFUml^-1^) was used to drench soil as described earlier [2] twice at weekly interval and the data was recorded after 28 days post CMV inoculation.

Measurement of morphological parameters from 12 plants per treatment was recorded at 28 dpi following the method of Nautiyal et al. [2]. Systemic leaves (fourth leaf from top) from three biological replicates (pooled from 4 plants per biological replicates) were collected, frozen immediately in liquid nitrogen, and used for further experiments. Leaf tissue, if not used immediately, was stored at -80°C until used.

### Disease severity index

Disease severity was evaluated by visual observation of systemic leaf (fourth fully expanded leaf from the top) following a rating scale of 0 to 3, on which 0 = no symptom; 1 = mild green mosaic symptom sparsely distributed on leaf surface; 2 = dark-green mosaic symptom spreader over 50% leaf area accompanied with leaf distortion and stunting; and 3 = severe dark-green mosaic symptom on whole leaf, leaf distortion, leaf narrowing, and severe plant stunting symptoms. Mock (buffer) inoculated plants were used as a control for each treatment set. Measurement of physical parameters was taken at 28 dpi of virus inoculations following the method described earlier [2]. Systemic leaf (fourth leaf from top) was harvested from each plant and frozen immediately in liquid nitrogen, and used for total RNA isolation for semi-quantitative PCR and quantitative real time-PCR (qRT-PCR).

### Enzyme-Linked Immunosorbent Assay (ELISA)

ELISA was performed to determine the relative level of coat protein (CP) accumulation in third leaf from the top from five plants of all four treatments: Control, B-30488, CMV, and B-30488+CMV plants at 28 days post virus inoculations (dpi). The 50 mg of leaf sample was independently ground in 50 mM carbonate buffer (1:10; w/v) using chilled mortar and pestle and supernatant was loaded onto ELISA 96-well plate. Coating of antigen (crude sap) to ELISA plate was done at 4°C overnight. Unbound antigen was washed away using PBS-Tween washing buffer (0.5% Tween 20 in PBS). The plates were then blocked with 5% nonfat dry milk in PBS-Tween containing 2% PVP (polyvinyl pyrollidone 40K, w/v) at 37°C for 1 h and washed five times. The primary antibody rabbit anti-CMV (PVAS 242a, ATCC, USA) (1:500) was incubated with antigen in the 96-well plates at 37°C for 1 h. After incubation, the plates were washed five times and secondary antibody goat anti-rabbit immunoglobulin conjugated to alkaline phosphatase (1:10,000) was added to each well, followed by incubation at 37°C for 1 h. Plates were washed five times to remove unbound secondary antibody and color reaction was developed at room temperature for 30 min using substrate (p-nitrophenylphosphate at l mg/ml in 10% diethanolamine, pH 9.8) (Sigma-Aldrich, USA). Finally reaction was stopped by adding 3N NaOH and plates were read (OD) using ELISA reader (BioRad model 550, USA) at A405 nm. Samples were considered positive for presence of CMV if the absorbance value exceeded three times of a threshold value equal to the mean of the absorbance value of healthy control samples.

### *In-vivo* detection of H_2_O_2_ in tobacco leaves

3,3’-diaminobenzidine (DAB) was used for the detection of H_2_O_2_ staining in tobacco leaf tissues. Briefly, the leaves were excised from all 4 treatments (Control, B-30488, CMV and B-30488+CMV plants) and floated in 0.1% (v/v) TritonX-100 containing 1 mg ml-1 DAB, and vacuum infiltrated for 30 min. DAB incubation was continued for 24 h. At the end of the staining, leaves were washed in distilled water, boiled in 95% ethanol for 10 min and examined for staining. H_2_O_2_ was visualized as a reddish brown coloration. The intensity of the coloration and its localization was qualitatively assessed and photographed.

### Evaluation of biophysical parameters in tobacco plants

#### Membrane ion leakage

The ion leakage of systemic leaf tissue of tobacco plants (collected from all four treatments: Control, B-30488, CMV, and B-30488+CMV) was measured following the method described earlier [28]. Leaves were immersed in 10 ml 0.4 M mannitol solution at room temperature with 200 rpm shaking for 3 h, and the bathing solution was measured for conductivity with a conductivity meter (Milwaukee Economical Pocket Tester, Noida, India). The total conductivity was determined by boiling leaves for 10 min. The conductivity due to leakage was expressed as the percentage of the initial conductivity versus the total conductivity.

#### Photosynthetic activity

Photosynthetic activity of leaves of tobacco plants was calculated following the method of Fan et al. [28] where the ratio of variable chlorophyll fluorescence (Fv) to maximum yield of chlorophyll fluorescence (Fm) was expressed as Fv/Fm. Leaves were dark-adapted in leaf clips of the fluorometer for 10 min at room temperature prior to activity measurements. The Fv/Fm ratio was recorded with a Pocket PEA Chlorophyll Fluorimeter (PP Systems International, Inc., MA, USA).

#### Chlorophyll (Chl) pigment estimation

Chlorophyll was extracted from tobacco leaf using 80% acetone as solvent. Briefly, 0.3 g of fresh leaf sample was homogenized in 3.0 ml of 80% chilled acetone (v/v). The absorbance was taken at 645 nm and 663 nm in a UV-160 spectrophotometer for Chl a and Chl b respectively, and chlorophyll contents (mg g^-1^ FW) were calculated using the equations suggested by [29] as

$$\text{Chl a }=\;\left(11.75\;\times {\text{ A}}_{663}-\;2.35\;\times {\text{ A}}_{645}\right)\times \text{ V}/\left(1000\;\times \text{ w}\right)$$

$$\text{Chl b }=\;\left(18.61\;\times {\text{ A}}_{645}-\;3.96\;\times {\text{ A}}_{663}\right)\;\times \text{ V}/\left(1000\;\times \text{ w}\right)$$

Where,

A_645_ = absorbance at a wavelength of 645 nm;

A_663_ = absorbance at a wavelength of 663 nm;

V = final volume of 80% acetone (ml);

w = dry weight of sample taken (g)

### Evaluation of biochemical parameters in tobacco plants

#### Determination of sugar, proline and Malondialdehyde

Total sugar (non-structural carbohydrate) estimation was carried out following an improved method [30]. Briefly, 0.2 g of fresh tobacco leaf tissue was crushed in 80% methanol. The extract was centrifuged at 10000 rpm for 10 min and obtained supernatant was incubated in water bath at 70°C. To this supernatant, equal volumes of 5% phenol and 5 ml of H_2_SO_4_ were added and absorbance was measured at 640 nm. A standard curve was prepared using different concentration of standard glucose solution and estimation of total sugar content was done on background of this graph.

Total proline content was quantified by the acid-ninhydrin procedure [31]. The leaf tissue (500 mg) was ground in 10 ml 3% sulfosalicylic acid and clarified by centrifugation. 2 ml supernatant was mixed with equal volumes of acid-ninhydrin and acetic acid, and the mixture was oven-incubated at 100°C for 1 h. Reaction was finished in an ice bath and extracted with 4 ml of toluene using a vortex mixer for 15–20 s and absorbance was measured at 520 nm.

The leaf malondialdehyde (MDA) content was determined following the protocol described earlier [32]. Briefly, three leaf samples were collected from each treatment; each sample of 0.3 g fresh weight was homogenized in 5 ml of 5% trichloroacetic acid. The homogenate was centrifuged at 8000 x g for 15 min. 1 ml supernatant of each sample was then mixed with 2.5 ml of thiobarbituric acid and heated at 100°C in a water bath for 20 min and quick chilled on ice. The mixture was centrifuged at 10000 x g for 5 min and absorbance of the resulting supernatant was measured at 532 nm and 600 nm. By subtracting the non-specific absorbance at 600 nm, the MDA content in leaves was determined by its molar extinction coefficient (155 mM^-1^ cm^-1^) and expressed as μmol MDA g^-1^ fresh weight.

#### Superoxide dismutase activity

Superoxide dismutase (SOD) activity was determined according to method of Beauchamp and Fridovich [33]. The reaction mixture containing 13 mM methionine, 2 mM riboflavin, 0.1 mM EDTA and 75 μM nitrobluetetrazolium (NBT) salts was dissolved in 3 ml of 50 mM sodium phosphate buffer (pH 7.8) to which 100 μl of SOD enzyme extract was added. The mixtures in glass test tubes were illuminated by Philips 40-W fluorescent tubes in triplicates and absorbance was read at 560 nm in the spectrophotometer against the blank. SOD activity is expressed in U mg^–1^ protein (U = change in 0.1 absorbance h^–1^ mg^–1^ protein under assay conditions).

#### Catalase activity

Catalase (CAT) activity was assayed according to the method of Chandlee and Scandalios [34]. The assay mixture contained 2.6 ml of 50 mmol l^–1^ potassium phosphate buffer (pH 7.0), 0.4 ml of 15 mmol L^–1^ H_2_O_2_, and 0.04 ml of catalase enzyme extract. Changes in absorbance were read at 240 nm. The enzyme activity was expressed in protein (U = 1 mM of H_2_O_2_ reduction min^–1^ mg^–1^ protein). The enzyme protein was estimated by the method of Bradford [35] for all the enzymes.

#### Guaiacol peroxidase activity

Guaiacol peroxidase (GPX) activity was assayed using the method of Zheng and van Huystee [36] by recording an increase in the absorbance at 470 nm as a result of oxidation of guaiacol to tetraguaiacol. The 1% guaiacal (v/v) and 0.3% H_2_O_2_ were dissolved in 50 Mm phosphate buffer (pH 6.6) to prepare a reaction mixture in a final volume of 3 ml. The linear portion of the activity curve was used to express enzyme activity (expressed as U mg-1 protein). One unit of GPX activity represented the amount of enzyme catalysing the oxidation of 1 μmol of guaiacol min^-1^.

#### Ascorbate peroxidase activity

Ascorbate peroxidase (APX) activity was determined spectrophotometrically by recording the decrease in absorbance at 290 nm because of oxidation of ascorbate in 3 ml of reaction mixture containing 50 Mm phosphate buffer (pH 7.0), 0.1 mM H_2_O_2_, 0.5 mM sodium ascorbate, 0.1 mM EDTA and a suitable amount of enzyme extract [37]. One unit of APX activity was assumed as the amount of the enzyme which oxidized 1 μmol ascorbate min^-1^ at 30°C.

#### Acid and alkaline phosphatase activity

Acid phosphatase (ACP) and alkaline phosphatase (ALP) activities were estimated from the root zone soil [38]. Briefly, 100 mg of soil sample was taken in a micro-centrifuge tube to which 0.5 ml of 100 mM phosphate buffer was added. 10 mM of p-nitro phenyl phosphate in 100 μl solution was used as substrate. The final volume of the reaction mixture was adjusted to 1 ml with the addition of requisite amount of distilled water. The tube was vortexed at room temperature for 2 min and incubated at 37°C for 60 min with 100 rpm shaking. Then, the sample was centrifuged at 10000 rpm for 5 min and clear supernatant was taken in a clean test tube and 2 ml of 1 M NaOH was added. The yellow colored filtrate was analyzed at absorbance 430 nm using a colorimeter (Systronics, India). Goat liver phosphatase activity was also determined as a reference using the same conditions.

### Rhizosphere microbial diversity analysis of tobacco using carbon source utilization pattern

Biolog Eco plates (Biolog, Inc., Hayward, CA, USA) were used to determine the carbon source utilization pattern in rhizosphere (soil) samples collected from plants of aforesaid four treatments as described earlier [39]. Data were recorded for 7 days at 590 nm and the result obtained at 3rd day was used for statistical analysis. Microbial activity in each microplate expressed as average well color development (AWCD). Diversity and evenness indices, principal component analysis were performed as described earlier. Statistical analyses were performed using SPSS 16.0 and Statistica 7.0.

### In vivo histological assay

For histology, hand cut sections of tobacco stem cross sections (CS) and leaf from all the four treatments were examined for changes in tissue anatomical features. Samples were fixed and rehydrated in FAA solution (formaldehyde:aceticacid:alcohol::5:5:90; v/v) for a week and then preserved in alcohol-glycerol mixture (1:1 mixture of 70% ethyl alcohol and glycerol). The washed samples were hand sectioned using new blades. The sections were stained using Toludine blue O (TBO) dye prepared by dissolving 1.0 g 1.0 g toluidine blue in 1% sodium borate in distilled water. TBO is an aqueous blue coloured cationic dye that binds to negatively charged groups generating different colours when bound with different anionic groups in the cell. Carboxylated polysaccharides such as pectic acid appears pinkish purple in color, polyphenolic substances such as lignin and tannins stain green, greenish blue or bright blue, nucleic acids show purplish or greenish blue appearance [40]. Photographs were taken on Olympus CX1 microscope fitted with Olympus digital camera (Leica Microsystems, GmbH, Germany).

### RNA extraction, cDNA synthesis and semi-quantitative PCR analysis

Total RNA was isolated from 100 mg leaf tissue of tobacco plants using TRIzol reagent (Invitrogen Co., Carlsbad, CA, USA) following the manufacturer’s instructions. All RNA preparations were treated with RNase-free DNase (Promega Co., Madison, WI, USA) to eliminate DNA contamination, assessed for quality before using for cDNA synthesis.

For cDNA synthesis, reverse transcription reaction was set up in a final volume of 20 μl using 5 μg of RNA, 200 U of RNaseH (Invitrogen Co. Carlsbad, CA, USA), 100 ng CMV-CP reverse primer (Table A in S1 File), 0.2 mM dNTPs and M-MuLV Revese Transcriptase enzyme (Invitrogen Co. Carlsbad, CA, USA). The cDNA synthesis was done following the manufacturer’s instructions.

Semi-quantitative PCR was performed in a final volume of 75 μl using 1.5 μl of cDNA, 1X PCR buffer, 1.5 mM MgCl_2_, 0.2 mM dNTP, 200 nM of each gene specific primers (Table A in S1 File) and 1 U of *Taq* DNA polymerase (Promega Co., Madison, WI, USA). The tobacco elongation factor 1alpha (EF1α) gene was used as internal reference gene for normalization of semi-quantitative-RT-PCR [41] and the amplification conditions were followed as suggested [42]. The amplified products were separated on 1.2% agarose gel to determine the level of expression of target gene. The intensities of the PCR bands were analyzed and quantified using Gel Doc 2000 and Quantity One Version 4.2.1 (Bio-Rad, USA) and image of ethidium bromide-stained agarose gels was acquired with Ultra-Lum CCD camera (Claremont, CA, USA). The relative intensities were calculated in reference to internal control (EF1α) and values were plotted by considering untreated control (C) as 100%.

### Quantitative RT-PCR (qRT-PCR) based expression analysis of genes related to development, stress and pathogenesis in response to B-30488 inoculation

Total RNA was extracted from tobacco leaves following the aforesaid procedure and treated with RNase-free DNase to eliminate genomic DNA contamination. cDNA was synthesized using the AccuScript High Fidelity 1^st^ Strand cDNA Synthesis Kit (Agilent Technologies, Santa Clara, CA, USA) following the manufacturer’s instructions. Primers sets targeting the genes related to development, stress and pathogenesis were used for expression analysis (Table B in S1 File). SYBR Green Master Mix (Agilent technologies, Santa Clara, CA, USA) was used for qRT-PCR and obtained data were analyzed using the delta-delta-Ct method [43]. *N*. *tabacum* Elongation factor 1-alpha (EF1α) was used as an internal control [41]. The following protocol was used for amplification: 94°C for 5 min followed by 35 cycles at 94°C for 30 s, 58°C for 30 s and 72°C for 60 s. The specificity of the individual PCR amplification was checked using a heat dissociation curve from 55 to 95°C following the final cycle of the PCR.

### Data Analysiss

The data were subjected to an analysis of variance using SPSS software (SPSS Inc., Illinois, USA). When a significant F test was obtained at *p* = 0.05, the separation of the treatment means was accomplished by Fisher’s protected LSD.

## Results

Biocontrol efficacy of B-30488 against CMV was determined in tobacco cv. White Burley plant under glasshouse condition. Here, the effect of B-30488 supplementation (drench on to pot soil) on tobacco plant vigour improvement and enhanced resistance ability to virus was validated by the measurement of physical, biophysical, biochemical, molecular and histological attributes and the findings are discussed.

### Disease severity in tobacco plants due to CMV infection

**Fig 1:**
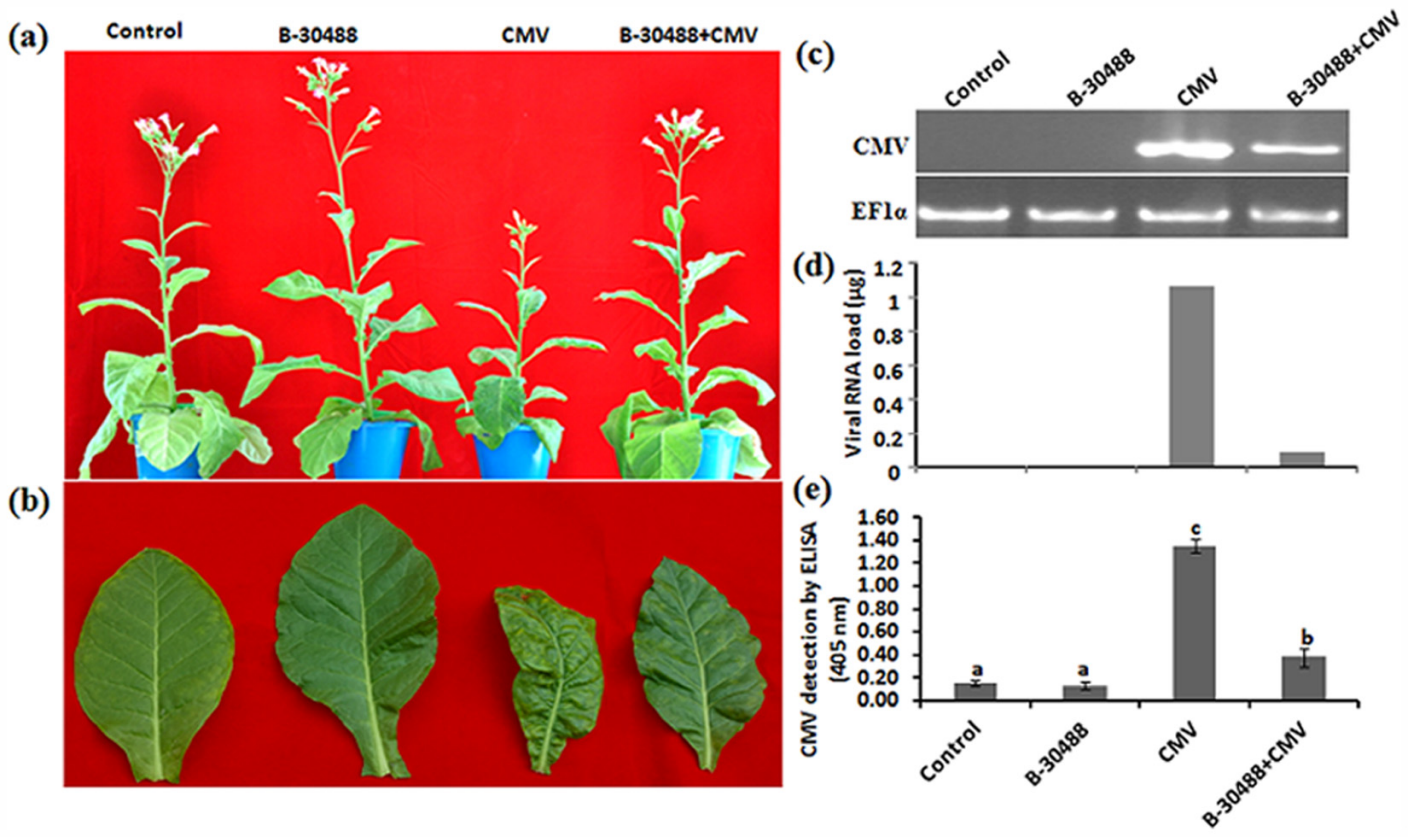
PGPR promoted the tobacco health and reduced virus load. (a) *N*. *tabacum* cv. White Burley plants showing induced growth at 28 dpi in B-30488 treated plant with or without CMV infection as compared to healthy (control) and disease (CMV) plants. (b) A close view of 3^rd^ tobacco leaf from top showing disease severity and chlorophyll differences. (c) Ethidium bromide stained agarose gel image showing high virus accumulation in leaf of CMV infected plant (CMV) as compared to B-30488 protected plant (B-30488+CMV). (d) Graphical representation of viral RNA load suggesting high CMV accumulation in infected tobacco (CMV) plant as compared to B-30488 protected plant (B-30488+CMV). Concentration of viral RNA was quantified by semi-quantitative PCR using CMV-coat protein (CP) gene specific internal primers and tobacco elongation factor 1alpha (EF1α) gene was used as an internal plant control. (e) Enzyme linked immunosorbent assay (ELISA) of upper leaves showing virus accumulation levels at 28 dpi. Plants were challenged with inoculum diluted ten times prepared from the leaves of tobacco infected with *Cucumber mosaic virus* (CMV)-A. Bar lines on each histogram indicate the standard error.

CMV inoculation induced foliar mosaic symptom in tobacco plants by 20 dpi which were prominent by 28 dpi (the day on which symptoms were observed) and have 2–3 scale disease rating on disease severity index of 3. Infected plants were exhibiting the symptoms of severe mosaic on more than 50% surface area of systemic leaves accompanied with leaf deformation and stunting of plant (Fig 1a, CMV) as compared to the healthy ones (Fig 1a, Control) which has 0 scale disease rating. Tobacco plants twice drenched with B-30488 (at one week interval) when challenged with high CMV inoculum pressure showed significant tolerance to CMV-infection as compared to non-bacterized CMV-infected plants besides showing the improvement in plant virgour. B-30488+CMV plants also showed 0–1 scale disease rating only at disease severity index of 3 (Fig 1a and 1b, B-30488+CMV) and the mosaic symptoms induced because of CMV-infection were very sparse and were suggestive of extenuation of CMV (Fig 1b).

### RT-PCR and ELISA

Molecular verification by RT-PCR with *CMV-CP* gene specific primers also confirmed the presence of CMV in systemic leaves (Fig A in S1 File), whereas failed to detect the virus in healthy plants confirming the symptoms were due to CMV infection. B-30488+CMV plants when quantified for virus RNA accumulation in systemic leaf, they showed lower amplification of *CMV-CP* (Fig 1c) suggesting low viral RNA accumulation. As expected, high accumulation of CMV RNA (represented by intense band) was observed in CMV-infected control plant. Band intensity based graph also corroborate with the results of qPCR and revealed the ~12 fold (91%) decrease in CMV RNA accumulation in B-30488+CMV plants as compared CMV-infected plants (Fig 1d). Molecular analysis suggested that the improvement in tolerance ability of tobacco plants against CMV infection may be due to soil supplementation of B-30488.

For the quantitative measurement of amount of CMV CP, ELISA of third leaf from the top of tobacco plants from all four treatments was done. ELISA revealed that the healthy control and B-30488 treated plants did not show any virus accumulation while, virus accumulation in B-30488+CMV plants was found ~3 fold lesser as compared to CMV inoculated plants (Fig 1e).

### B-30488 promote plant growth while mitigate CMV impact in tobacco plants

Morphological analysis showed significant reduction of 30.4, 12.5, 6.5, 19.0, 14.2 and 17.7% in shoot length, root length, shoot thickness, number of leaves, fresh weight and dry weight, respectively was noticed in infected plants as compared to the healthy plants (Table 1). They also bore 25.4% lesser flowers and perceptibly lesser seeds. Collectively, noticeable losses in plant vigour, flowering and seed setting was observed due to CMV infection. Soil supplementation of B-30488 substantially increased tobacco plant vigour (Fig 1a, B-30488) as compared to the control ones (Fig 1a, Control). Bacterized plants showed increase of 37.8, 36.4, 73.1, 19.0, 42.4 and 33.6% in shoot length, root length, shoot thickness, number of leaves, total plant fresh and dry weight, respectively (Table 1). They also showed 186.9% increase in number of flowers and perceptibly higher number of seeds than in control ones. B-30488+CMV plants showed noticeable increase of 59.8, 78.3, 91.7, 44.1, 163.0, 82.3 and 64.7% in root length, shoot length, shoot thickness, number of leaves, number of flowers, total plant fresh and dry weight, respectively which was clearly comparable to CMV-infected tobacco plants (CMV) drenched with growth media only (Table 1).

**Table 1:** Plant growth parameters in control, B-30488 (*P*. *lentimorbus* B-30488), CMV-infected and B-30488+CMV-infected *N*. *tabacum* cv. White Burley plants by analyzing the physical parameters (in their respective measurement units).

Treatments	Shoot length (in cm)	Root length (in cm)	Shoot thickness (in mm)	Fresh weight (in g)	Dry weight (in g)	Number of leaves	Number of flower
Control	15.67±0.88^b^	6.67±0.45^b^	7.03±0.58^ab^	18.27±0.54^b^	1.07±0.12^b^	14.00±1.15^b^	17.00±1.15^b^
B-30488	21.60±0.90^d^	9.10±0.36^d^	12.17±0.44^c^	28.03±2.83^d^	1.43±0.12^c^	16.67±0.67^c^	48.67±0.88^d^
CMV	10.90±1.16^a^	5.83±0.17^a^	6.57±0.41^a^	15.67±0.47^a^	0.88±0.04^a^	11.33±0.88^a^	12.67±0.88^a^
B-30488+CMV	18.00±0.80^c^	8.87±0.26^c^	12.60±0.21^c^	26.57±0.78^c^	1.45±0.07^c^	16.33±0.88^c^	33.33±0.67^c^

The data was recorded at 28 dpi. Different letters (a-d) showing significant difference at *P* = 0.05 using Waller—Duncan test.

### Effect of B-30488 on biophysical and biochemical attributes

**Fig 2:**
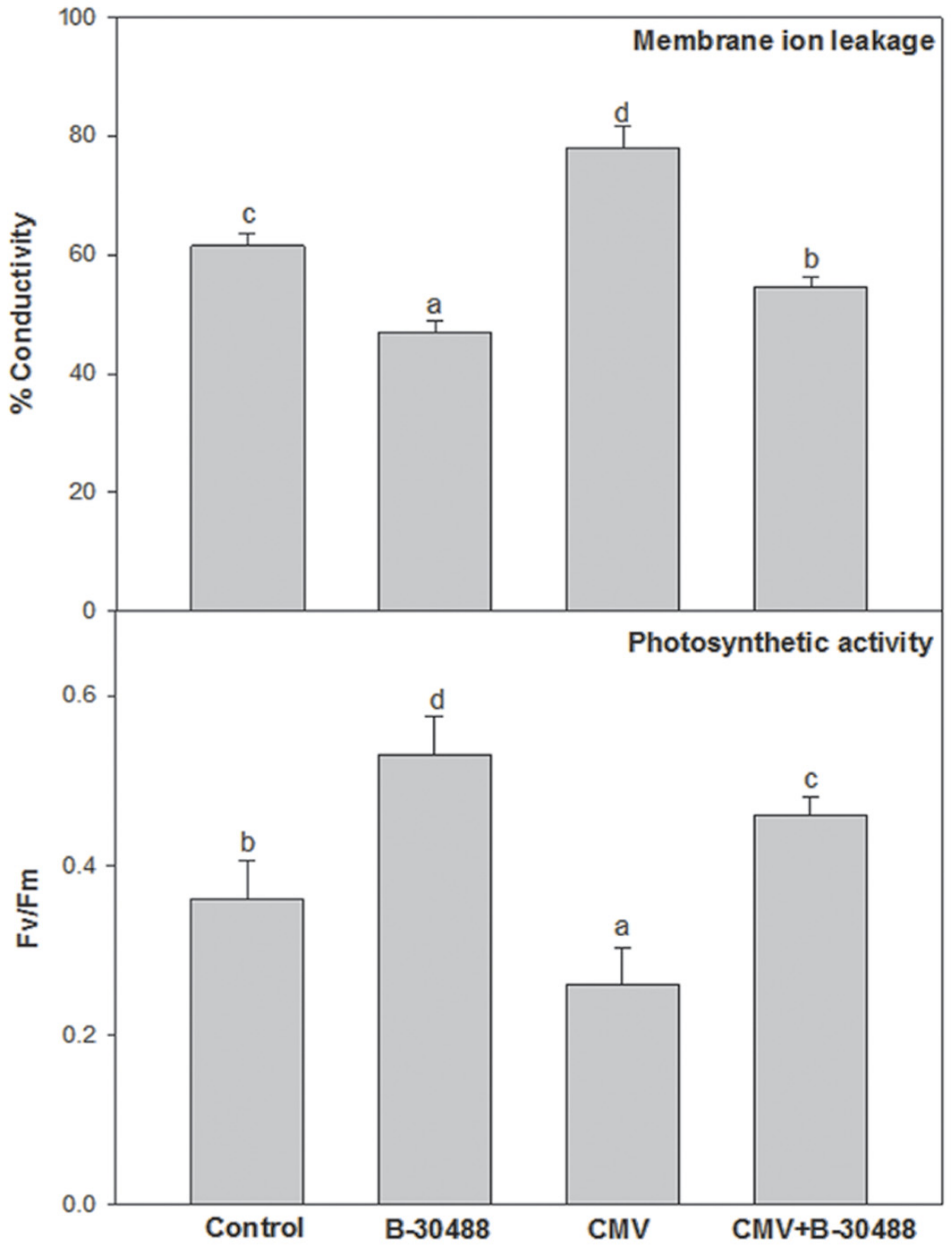
B-30488 inoculation improved the biophysical characters in tobacco plants. Graphs for biophysical (membrane ion leakage and photosynthetic activity) in systemic leaf of *N*. *tabacum* cv. White Burley plant collected from each treatment (Control, B-30488, CMV and B-30488+CMV) revealing the improved plant efficiency in bacterized plant. The data was calculated at 28 dpi.

Significant increase of plant vigour and induced tolerance to CMV due to B-30488 encouraged us for further study of biophysical (membrane ion leakage and photosynthetic activity) and biochemical (chlorophyll, sugar, proline and MDA radicals) attributes in systemic leaf tissue of tobacco plants. These attributes have clear correlations with the plant stress. B-30488+CMV plants showed significant (48%) reduction in membrane ion leakage as compared to the CMV-infected plants. Likewise, bacterization improved the photosynthetic efficiency (Fv/Fm) of both CMV-infected and non-infected plants by 75 and 47%, respectively (Fig 2).

**Fig 3:**
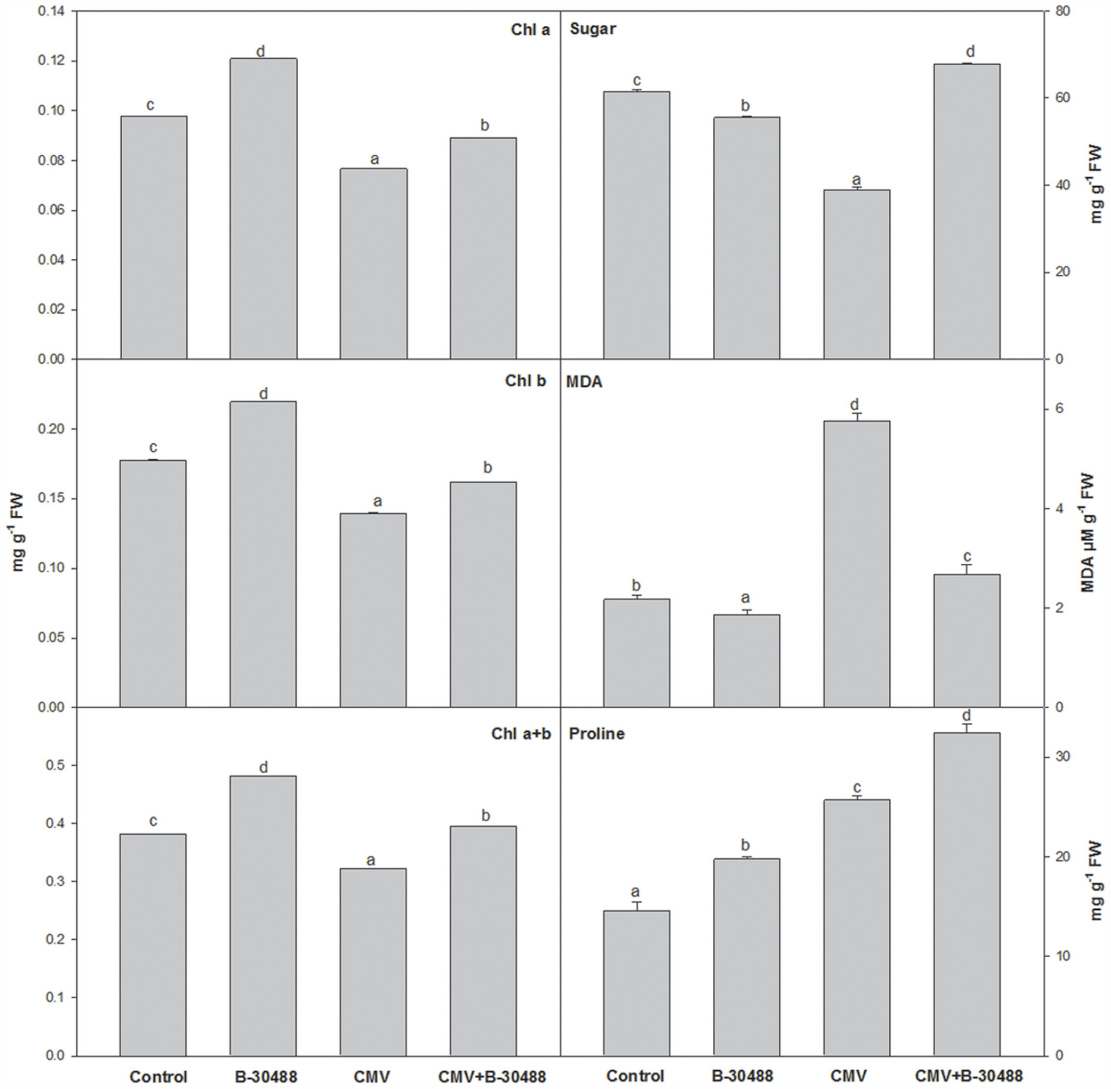
B-30488 inoculation improved the biochemical characters in tobacco plants. Graphs for biochemical (chlorophyll, sugar, proline and MDA) characters in systemic leaf of *N*. *tabacum* cv. White Burley plant collected from each treatment (Control, B-30488, CMV and B-30488+CMV) revealing the improved plant efficiency in bacterized plant. The data was calculated at 28 dpi.

The assessment of biochemical characters revealed that soil supplementation of B-30488 enhanced chl a, chl b and total chlorophyll contents by 23.7, 23.7 and 25.9%, respectively in B-30488 plants as compared to control ones. Reduction of chl a, chl b and total chlorophyll content was noticed in CMV-infected plants by 21.7, 21.6 and 15.7% respectively, which was ameliorated by 16.2, 16.3 and 22.8% respectively in B-30488+CMV plants (Fig 3). On the other hand, soil supplementation of B-30488 reduced the leaf MDA content by 14% (lowered from 2.2 μmol g^-1^ to 1.9 μmol g^-1^ concentration) in tobacco plants as compared to the control plants. The MDA content in CMV-infected plants was 5.7 μmol g^-1^ and soil inoculation of B-30488 lowered it by 54% (2.7 μmol g^-1^) in CMV-infected plants. The response of total soluble sugars in bacterized plants was not as consistent as observed with other parameters. B-30488 reduced the sugar content by 9.6% in B-30488 plants as compared to control ones, while it was increased by 10.2% in B-30488+CMV plants. Like total sugar, total proline content was also variable in B-30488+CMV, CMV and Control plants. B-30488+CMV plants showed 20.9% decrease in proline as compared to CMV-infected plants. On the other hand, inoculation of B-30488 increased proline content by 35.5% in B-30488 plants as compared to control ones (Fig 3).

### ROS accumulation in tobacco plants

In order to investigate the oxidant status and generation of ROS, in turn of H_2_O_2_ production, in leaf tissues of tobacco plants from all four treatments was assayed with the help of DAB which polymerizes and turns deep brown in the presence of H_2_O_2_. The development of the DAB-H_2_O_2_ reaction product in *N*. *tabaccum* leaves, in response to B-30488 and CMV treatments is shown in Fig B in S1 File. DAB staining assay indicated that the H_2_O_2_ production was concentrated in the whole leaf of CMV-infected plant while it was marginally appeared in control and PGPR treated plant leaves. The H_2_O_2_ production was observed less in leaf of CMV-infected plant which was ameliorated by B-30488. This finding proved the well developed theory of ROS production during virus infection.

### Effect of B-30488 inoculation on stress related enzymes

**Fig 4:**
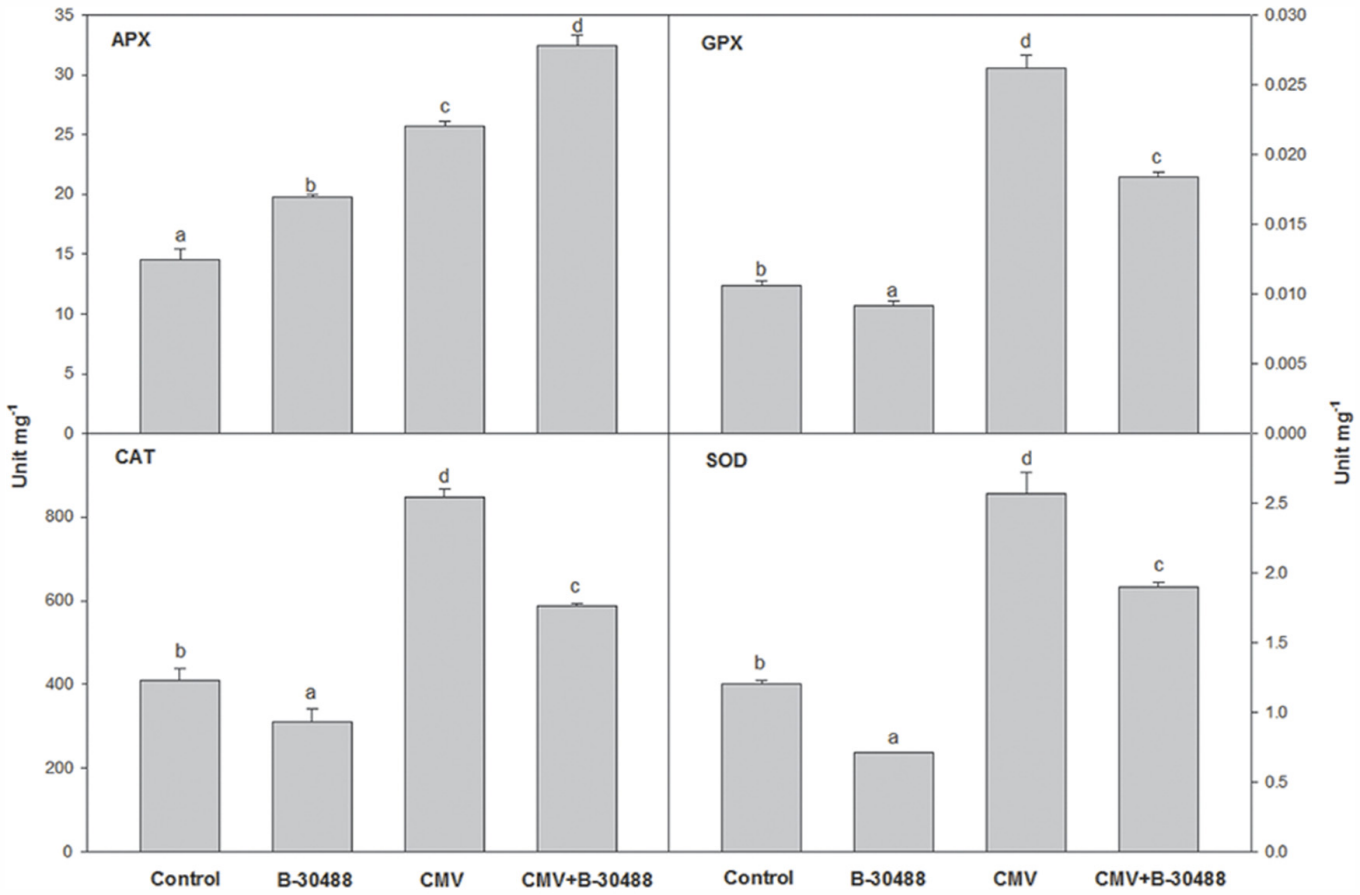
B-30488 inoculation improved defense system in tobacco plants. Graphs of defense related enzyme (SOD, APX, GPX and CAT) activity in systemic leaf of *N*. *tabacum* cv. White Burley plant collected from each treatment (Control, B-30488, CMV and B-30488+CMV) revealing the improved defense ability of bacterized plant against the CMV infection. The data was calculated at 28 dpi.

The physical, biophysical and biochemical parameters observed in B-30488 soil supplemented tobacco plants were supportive of their improved health and tolerance to CMV-infection, therefore, accumulation levels of selected stress related enzymes (SOD, CAT, APX and GPX) were assessed. Estimation of SOD, CAT, APX and GPX in B-30488 plants showed significantly reduced activity by 41.6, 23.9, 28.5 and 10.0% respectively as compared to control ones (Control). Biochemical analyses suggested that tobacco plants were in high spirits in pot soil drenched with B-30488 and, therefore, showed reduced activity of these enzymes. B-30488+CMV plants also showed 35.4, 30.6, 23.0 and 30.7% reduction in level of SOD, CAT, APX and GPX enzymes, respectively (Fig 4) as compared to CMV-infected plants. This study suggested the improvement in biotic stress tolerance of CMV-infected tobacco plants was due to soil supplementation of B-30488.

### qRT-PCR based expression analysis of development, pathogenesis and stress related genes

**Fig 5:**
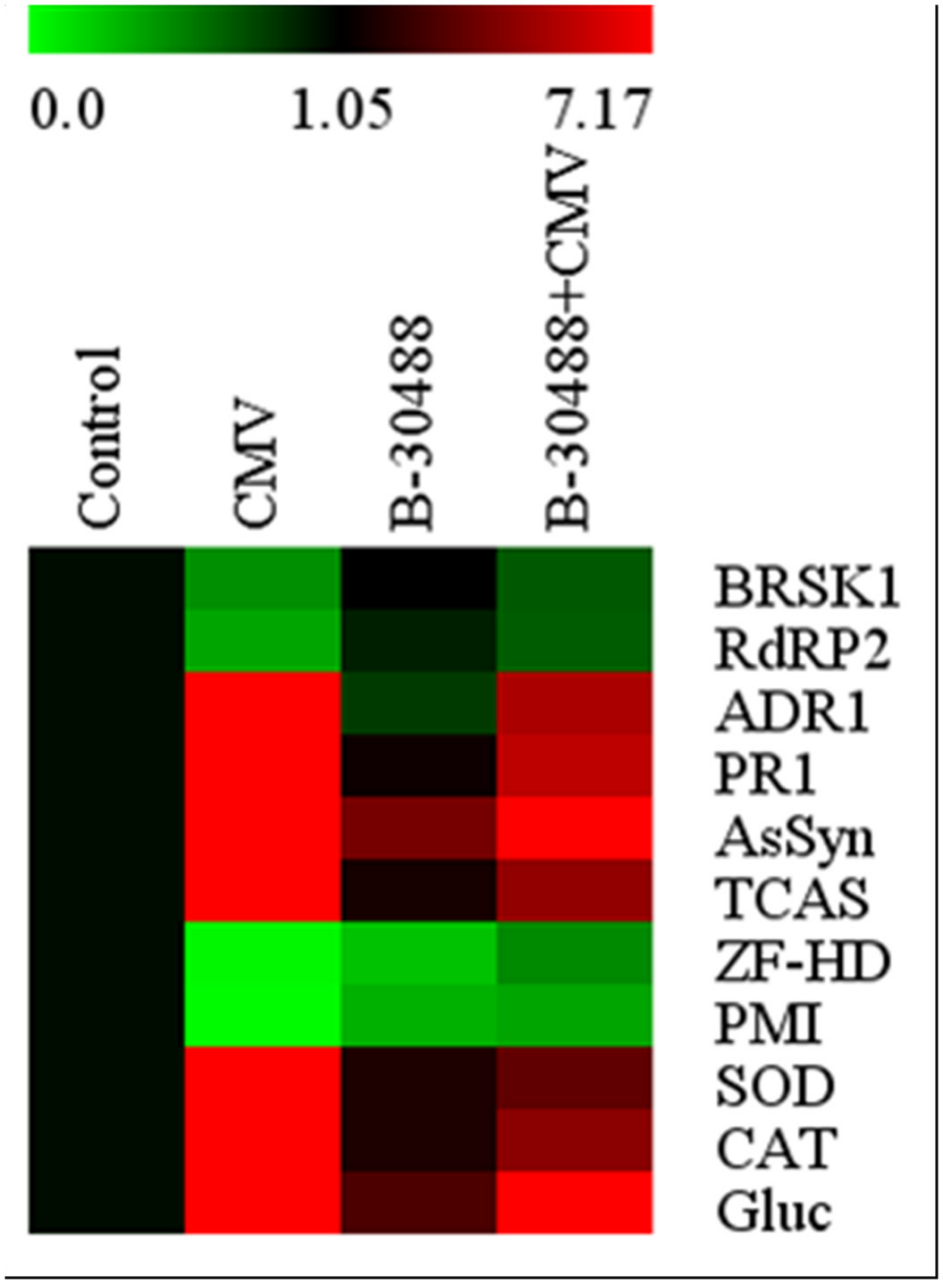
Quantitative validation of relative expression of selected transcripts related to development, stress and pathogenesis. Heat map of BR-SK1, RdRP2, ADR1, PR1, AsSyn, TCAS, ZF-HD, PMI, SOD, CAT and Gluc genes showing their relative expression by qRT-PCR. The signal intensity of each transcript was normalized using Actin (a house keeping gene). The top most bar represents the fold change expression values of selected genes.

Expression analysis of these genes very well corroborate with the results obtained with biochemical analyses. Soil supplementation of B-30488 (prior to CMV inoculation) significantly decreased the expression level of defense related genes in tobacco plants challenged with CMV (Fig 5). qRT-PCR analysis showed that genes encoding for ADR1, PR1, SOD, CAT, Gluc, AsSyn and TCAS were notably more highly expressed by 20.6, 21.4, 11.3, 7.1, 43.3, 43.7 and 15.1 fold respectively in CMV-infected tobacco plants compared to healthy leaves. Application of B-30488 significantly curtailed the expression of these genes by 5.1, 5.6, 3.4, 4.4, 22.8, 8.5 and 4.5 respectively. The suppression of ZF-HD homeobox and PMI protein genes by 0.02 and 0.01 fold respectively was noticed clearly in CMV infected plants which was restored by 0.4 and 0.3 fold in B-30488 inoculated plants. The expression of BRSK1 and RdRP2 genes was slightly down-regulated (by 0.4 and 0.3 fold respectively) in CMV infected plants and was significantly ameliorated (upregulated by 0.6 and 0.6 fold) in B-30488 inoculated plants. In B-30488 inoculated control (B-30488) plants, the expression of aforesaid genes were expectedly regulated (up or down) that support and favor plant’s health and immunity too.

### Effect of B-30488 on functional diversity of rhizosphere

Results obtained from carbon source utilization profiles of rhizosphere soil of Control, B-30488, CMV and B-30488+CMV-infected tobacco plants demonstrated the enhanced microbial diversity and related evenness (Table 2). As CMV-infection deteriorated the plant health and growth, the same was reflected for the associated rhizosphere. A significant reduction in the diversity and related evenness indices due to CMV-infection in tobacco plant rhizosphere was observed. Similarly, the effect of improvement in plant health due to B-30488 inoculation was manifested in rhizosphere microbial diversity.

**Table 2:** Diversity and Evenness index of tobacco rhizosphere treated with B-30488, CMV and B-30488+CMV, based on substrate utilization pattern Biolog Eco plates containing 31 different carbon sources.

Index	Control	B-30488	CMV	B-30488+CMV
Shannon diversity (ShD)	3.142±0.015^b^	3.326±0.016^c^	2.991±0.055^a^	3.314±0.010^c^
Shannon evenness (ShE)	0.915±0.004^b^	0.969±0.005^c^	0.882±0.010^a^	0.965±0.003^c^
McIntosh diversity (McD)	0.947±0.003^b^	0.982±0.002^c^	0.921±0.009^a^	0.980±0.002^c^
McIntosh evenness (McE)	0.947±0.003^b^	0.982±0.002^c^	0.926±0.006^a^	0.980±0.002^c^
Simpson diversity (SimpD)	0.982±0.001^b^	0.994±0.001^c^	0.972±0.004^a^	0.994±0.001^c^

± Standard error (n = 3); Different letters showing significant difference at *P* = 0.05 using Waller—Duncan test.

**Fig 6:**
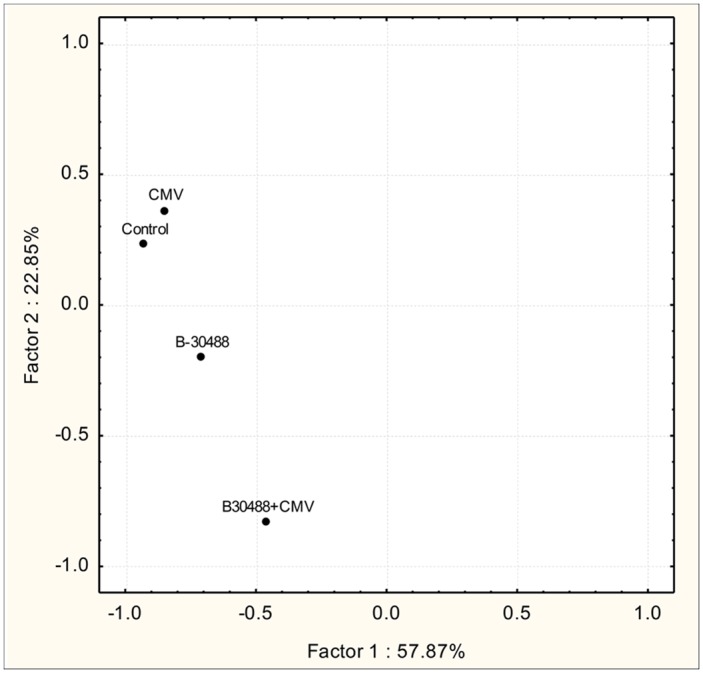
B-30488 enhanced the colonization of beneficial population in tobacco roots. Principal component analysis of carbon source utilization by rhizosphere microbial community of control; B-30488; CMV and B-30488+CMV tobacco plants using Biolog Eco Plates.

Principal component analysis from the carbon source utilization pattern of tobacco rhizosphere samples was distributed 57.87 and 22.85% on PC1 and PC2 axis (Fig 6). Results revealed that inoculation of B-30488 was responsible to alter the rhizosphere microbial population as compared to the non-bacterized control as well as CMV-infected plants. Control and CMV-infected plant rhizosphere microbial community were also separated clearly reflecting that virus infection had decreased the growth of plant which consequently decreased the microbial activities.

### Effect of B-30488 on ACP and ALP

**Fig 7:**
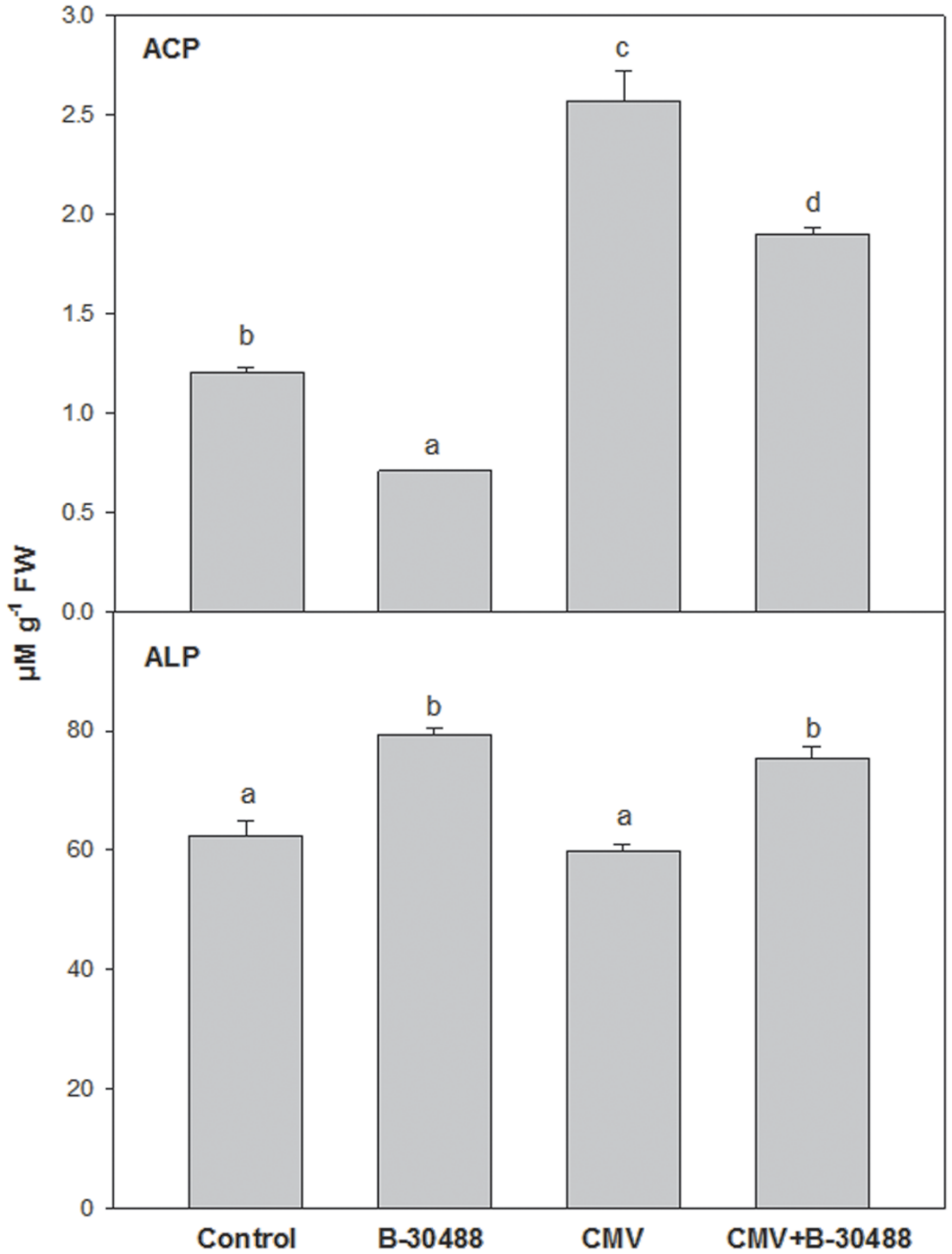
ACP and ALP activities also increased in B-30488 inoculated tobacco plants. Graphs of soil phosphatases (acid and alkaline phosphatase) in root rhizosphere of *N*. *tabacum* cv. White Burley plant taken from each treatment (Control, B-30488, CMV and B-30488+CMV) revealing the improved phosphate uptake efficiency in soil inoculated plant. The data was calculated at 28 dpi.

The ACP and ALP activities in tobacco root rhizosphere of tobacco plants from all four treatments were measured to assess the mineral phosphate solubilization efficiency from the pot soil. The soil supplementation of B-30488 improved soil inorganic phosphate uptake efficiency in control (B-30488) and CMV infected (B-30488+CMV) plants by 22.8 and 34.4%, respectively (Fig 7). The control and CMV-infected plants showed more or less similar enzyme activity.

### Effect of B-30488 on plant anatomy

**Fig 8:**
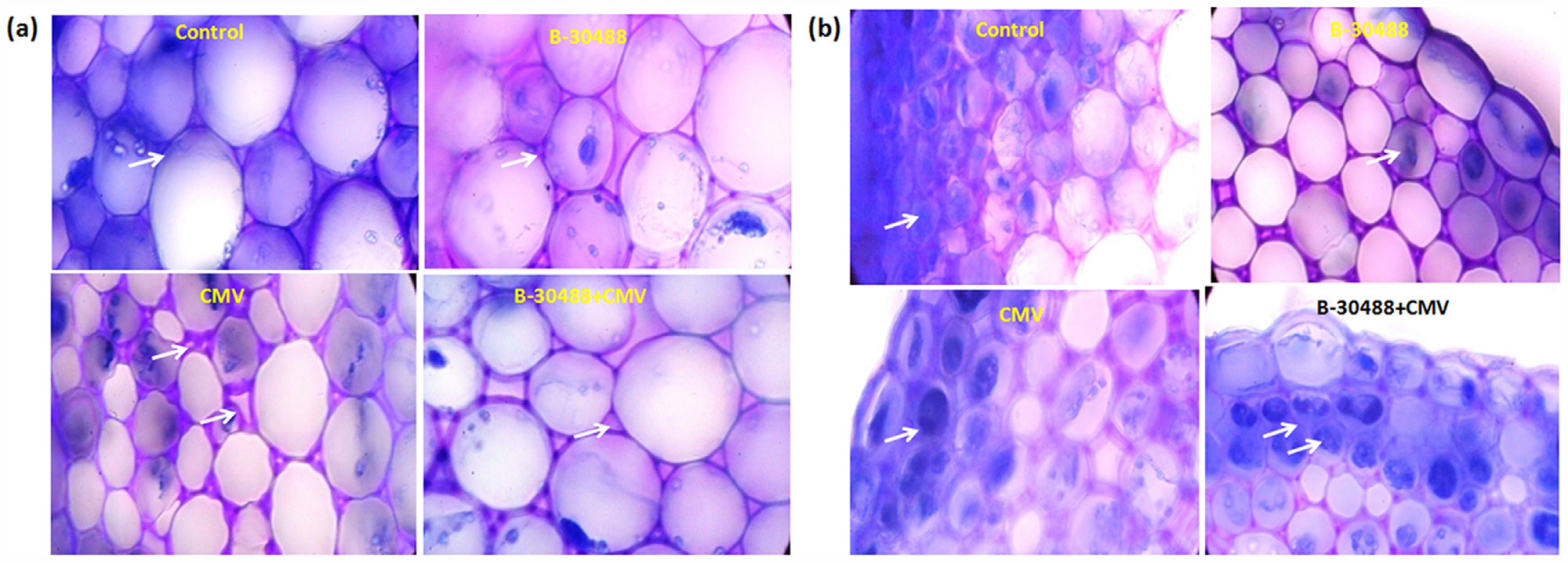
Hisology of stem tissue. Histological analysis of stem tissue of *N*. *tabacum* cv. White Burley plants showing effect of PGPR (*P*. *lentimorbus*, B-30488) in presence and absence of virus (CMV). (a) Healthy collenchyma cells are observed in control, B-30488 and CMV+ B-30488 treatments while CMV infection caused shrinkage of the cells altering the intercellular space (shown by the arrows) and thickened cell connecting corners (b) Stem tissue showing polyphenol accumulation in hypodermis layer (shown by arrows). All the pictures were taken at 100X magnification

Anatomical studies of stem (internode between the fourth and fifth leaf from the top) and systemic leaf tissue of tobacco plants from all four treatments were carried out to reveal the structural status of the cells. Stem CS of control plants revealed that the cells were in a well turgid state and in good health with least intercellular spaces among the collenchymas (Fig 8a). The cell’s connecting corners were normal without any thickness. Moreover, the polyphenol accumulation (a stress related defense enzyme) in hypodermis layer was normal (Fig 8b; Fig C in S1 File). These studies represented that control plants were surviving without any stress in glasshouse conditions. While, establishment of CMV-infection in tobacco plants resulted in morphological alterations. Intercellular spaces between collenchymas were increased and cells appeared to be loosely arranged with thickened and prominent cell connecting corners (probably due to the polymers like xylogalacans and pectins connecting the primary cells in aged or stresses cells) [44]. Besides this, the collenchymas appeared to be deflated as compared to the control ones (Fig 8a). The stem of B-30488 and B-30488+CMV plants showed turgid cells (in CS) as were observed in control plants with normal cell wall thickenings, however, the intercellular spaces of collenchymas did show comparatively wider intercellular spaces (Fig 8a).

**Fig 9:**
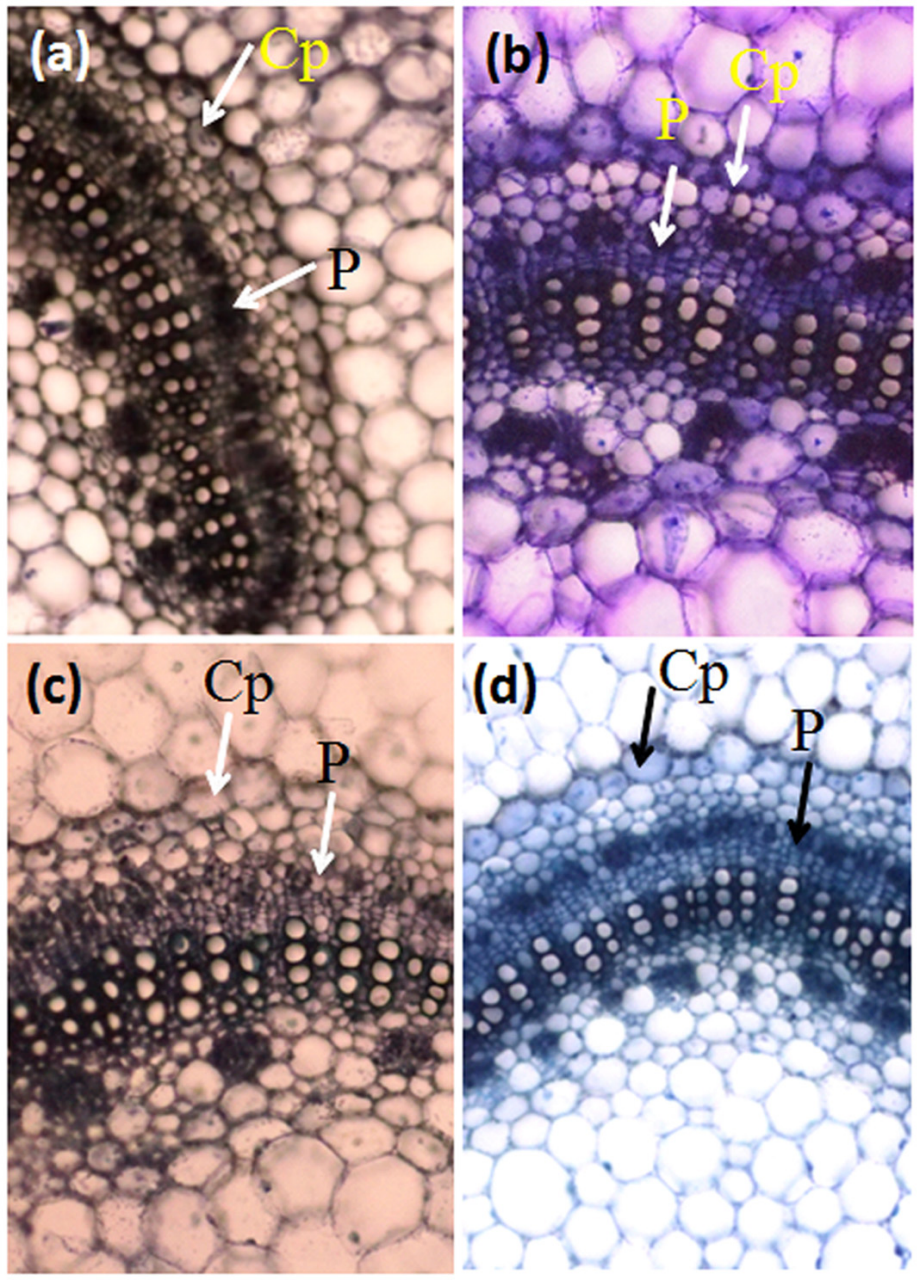
Hisology of tobacco leaf suggesting PGPR mediated tissue improvements. The leaf vasculature of (a) control (b) B30488 (c) virus infected (d) B30488 + virus treated plants showing relatively undifferentiated casparium (cp) and pericycle (p) in virus infected plants. All the pictures were taken at 10X magnification.

The vasculature of the CMV-infected leaf section revealed a relatively undifferentiated casparian stripe and pericycle as compared to well formed casparian band and pericycle in control and both the B-30488 treatments (Fig 9). Histopathological analysis of virus-infected and non-infected leaf and stem CS suggested that virus induced structural anomalies in the tissues which were negated in B-30488 primed plants infected with the virus.

## Discussion

The applicability of B-30488, a PGPR (isolated from native Sahiwal cow’s milk [25]) that has already shown the potential of enhancing plant vigour [7] and ameliorating the biotic and abiotic stress [5,7], has been further assessed as a BCA against virus (CMV) infection in *N*. *tabacum* cv. White Burley plants. Among the bacterial genera, *Pseudomonas* and *Bacillus* are the two most studied genera which have been known for enhancement of plant health and stress tolerance [1]; however, limited information exists on the deployment of PGPR for induced resistance against virus-infection. Earlier, Raupach and colleagues figured out that protection against CMV infection in cucumber and tomato plants may be achieved by employing *Pseudomonas fluorescence* 89B-27 strain as PGPR [24]. Later on, the protection of tomato plants against *Tomato mottle virus* was demonstrated by consortia of 3 PGPR stains: *Bacillus amyloliquefaciens* 937a, *B*. *subtilis* 937b and *B*. *pumilus* SE34 [12]. In another study, health improvement along with ISR attributes were shown in tobacco plants against *Tobacco mosaic virus* by application of consortia of *B*. *subtilis* G1 and B3, and *B*. *amyloliquefaciens* FZB24 and FZB42 [45]. Notably, the use of any *P*. *lentimorbus* strain for induced resistance against CMV infection has not been investigated. Present study revealed that soil drenched with B-30488 significantly diminished the disease severity rating from 2–3 to 0–1 scale on disease severity index of 3 and reduced CMV RNA accumulation level by ~12 fold in systemic leaves as compared to that of CMV-infected plants. Moreover, B-30488 compensates the morphological losses caused by CMV-infection to a significant extent (Figs 8 and 9) and augmented the tobacco plant vigour, the atypical PGPR attributes studied earlier [7,25].

The low level of CMV accumulation in systemic leaves of B-30488+CMV plants (~3 fold) than the diseased control plants, assessed by ELISA, was suggestive of protection against the CMV due to PGPR. Protection against CMV by B-30488 treatment appeared 20–75% elimination of viral symptoms. The study very well corroborate with the earlier findings where protection of different crops by PGPR treatment against different viruses was achieved. Earlier findings with CMV suggested that PGPR treatment was able to either completely eliminate development of viral symptoms or to reduce disease severity in cucumber, tomato, and Arabidopsis [13,24,46]. Similarly, ELISA result showed lower CP amount of TMV and Tomato mottle virus (ToMoV) in PGPR treated tobacco [47] and tomato [12] plants, respectively. Further, DAB staining assay also confirmed that H_2_O_2_ accumulation was observed in CMV-infected plant leaf while as compared to control and PGPR treated plant leaves. Moreover, the H_2_O_2_ production was observed less in leaf of CMV-infected plant which was ameliorated by B-30488 (Fig B in S1 File). This finding proved the well developed theory of ROS production during virus infection.

Both, biotic and abiotic stresses negatively affect the survival of plant, plant fitness and induce aging [48] which is determined by the measurements of stress induced biomolecules. Under stress conditions, the level of MDA (a resulting byproduct of lipid peroxidation and differentially regulated in thylakoid and plasma membrane) is often used to explain the extent of peroxidation and integrity of membrane system [49]. MDA has been reported to be induced during drought and heat as that the imposition of stress on plants leads to enhanced membrane peroxidation in leaf tissues. However, increase in MDA was also noticed in CMV-infected tobacco plants which were effectively curtained to 54.6% in CMV-infected plants by soil inoculation of B-30488. The other issue related with MDA is membrane ion leakage and is directly proportional to the level of MDA and, therefore, increase (by 23.8%) in leaf ion leakage was observed in CMV-infected plants which were effectively reduced by 48.5% in B-30488+CMV-infected plants, suggesting the improved structural integrity due to B-30488.

Chlorophyll is critical in photosynthesis for production of food in the form of carbohydrate. Lenin and Jayanthi [50] found that consortia of four PGPR strains (*Azospirillum lipoferum*, *Azotobacter chroococcum*, *P*. *fluorescens* and *B*. *megaterium*) increased the total chlorophyll content in Catharanthus plant by 5.34 mg g^-1^. In another study, Baset Mia et al. [51] also observe that inoculation of PGPR: *B*. *sphaericus* UPMB10 and Sp7 in banana plants raises the total chlorophyll content by 25 to 33% compared to non-bacterized ones. A close up of 3^rd^ top leaf showed the disease severity in terms of mosaic symptoms and visual difference in chlorophyll content (Fig 1b) which was further evidenced by biochemical assays. The assessment of biochemical characters revealed that soil inoculation of B-30488 enhanced chl a, chl b and total contents by 23.7%, 23.7% and 25.9%, respectively in control plants. Reduction of chl a, b and total content was noticed in CMV-infected plants by 21.7, 21.6 and 15.7%, respectively which was ameliorated by 16.2, 16.3 and 22.8%, respectively (Fig 2) by soil inoculation of B-30488. In our study, the influence of virus infection on chlorophyll content was noticeable in infected tobacco plants. The increased leaf chlorophyll content in B-30488 inoculated tobacco plants resulted in increased photosynthetic efficiency (Fv/Fm). Recently, Kandasamy et al. [52] have observed that priming of *Pseudomonas fluorescens* (PGPR), enhanced expression of Ribulose-bisphosphate carboxylase large chain precursor which plays a significant role in photosynthesis and accumulation of chlorophyll.

The regulation of proline is crucial for maintaining the osmotic potential of tissues [53]. Increase of proline content was observed in CMV-infected tobacco plants which may be due to disturbed electrolyte system in infected tissue and, to counter balance this, the level of proline was probably upregulated to maintaining the osmotic potential of leaf tissues. Studies have shown the role of PGPR mediated biotic stress amelioration of plants [54,55]. A stable and balanced intercellular redox is of vital importance to all organisms. A critical balance is maintained by the plant defense mechanism by differentially regulating various enzyme and/or metabolites to balance the ROS status. Modification of proline level by B-30488 and CMV in different treatments proved to act as indicator of ROS status in plant; lowered proline and ROS in B-30488 and higher in B-30488+CMV treatments shows the ability of the bacteria to maintain ROS balance as per requirement of plant. Need for proline in maintaining the elevated ROS level and shift in nitrogen metabolism has been reported earlier [56].

An induced level of ROS quenching enzyme such as SOD, CAT, APX and GPX in CMV treatment is indicative of a higher intercellular redox and is an indicator of stress. Induction of these defense enzymes have been reported in plant during the CMV infection [57]. On the other hand, reduced SOD, CAT, APX and GPX activity in B-30488+CMV infected plants shows a controlled intracellular redox. How this control is mediated is not known; however, it does indicate the ability of B-30488 to control the stress induced by CMV such that the ROS is balanced at cytosolic level. The observations are confirmatory in demonstrating the role of priming plants with B-30488 in balancing ROS level for prolonged growing period of the plant and inducing ISR during virus infection and provide protection.

The gene expression data obtained by qRT-PCR corroborate well with the data obtained with biochemical analyses. A clear upregulation of SOD and CAT genes along with ADR1, PR1, Gluc, AsSyn, TCAS genes involved in development, stress and pathogenesis [58–62] was observed in virus infected tobacco plants, which was significantly down regulated in those tobacco plants where B-30488 was soil supplemented prior to virus inoculation. The results obtained in this study are well supported by a previous study where upregulation of stress and pathogenesis related genes were observed in tobacco plants infected by M-CMV strain [62]. Together, biochemical and molecular studies are suggestive of amelioration of tobacco plants and suppression of virus induced stress in presence of B-30488.

Phosphatase is an enzyme that release inorganic phosphate from organic moiety and complex inorganic materials in soil and, therefore, the amount of phosphate released into the soil can then be directly co-related to soil fertility. Soil receives various phosphatases from living organisms that plays critical role in the process of solubilization of phosphate and bacteria are the main sources of phosphatases in a soil environment [63]. Strains from the genera *Pseudomonas* and *Bacillus* are among the most powerful phosphate solubilizers [64,65]. Enhanced production of both ACP and ALP enzymes in presence of B-30488 is suggestive of its high mineral phosphate solubilization activity.

Studies of rhizosphere structure and functioning have proved difficult because of the complexity of the soil. However, advances in the development of analytical tools such as Biolog, which permit microbial communities to be characterized according to their physiological profile quantified by a redox reaction due to the growth of inoculated microbial communities that changes color of the indicator dye nitroblue tetrazolium [66–69]. Microbial community structure in the rhizosphere of all the four treatments was assessed, using Biolog Eco plates which suggested that inoculation of B-30488 makes the maximum increase in the tobacco rhizosphere functional diversity. The Principal components (PC) score plots describe the characteristics of the samples and help to understand their spatial distribution and clustering on a PC plot. Principal component analysis (PCA) of carbon source utilization pattern on Biolog Eco plates did not make any cluster among the four samples, as they were distributed separately among each other at 57.87 and 22.85% on the PCA vector 1 and 2 axis. Rhizosphere from B-30488 and B-30488+CMV-infcted plants were found to be most separated, while control and CMV-infected could loosely be grouped together. Results indicate that in both cases, B-30488 and B-30488+CMV treated tobacco rhizosphere, there were maximum change in microbial community structure, compared with lesser changes in control and CMV-infected tobacco rhizosphere microflora.

Cumulatively, the present study delineated several biophysical and biochemical attributes by considering structural integrity (membrane ion leakage and MDA) the level of energy (sugar), photosynthetic activity (chlorophyll content and photosynthetic activity) and activities of stress related enzymes (proline, SOD, APX, GPX and CAT). However, pathways that maneuver the plant vigor as well as induced the defense machinery of tobacco plants needs further extensive molecular investigations. The application of B-30488 effectively increases the plant growth parameters and reduces the incidence of CMV and eventually improved the yield attribute in field conditions. Maximum increase in seed yield with an additional income and benefit cost ratio was recorded in B-30488 treatment. Hence, this formulation may be recommended after due field testing to the related farmers as one of the crop protection strategies for the management of CMV and this practice may also be extended to other crops and for other viruses too.

## Supporting Information

S1 File Supplementary figures and tables. **Table A:** Details of primers used for this study. **Table B:** List of selected primer pairs of *N*. *tabacum* used for real time-PCR. **Fig A: Detection of CMV in *N*. *tabaccum* cv. White Burley plants.** Agarose gel image showing ~650 bp amplification from leaf samples of CMV-infected *N*. *tabaccum* cv. White Burley plants confirming the presence of virus in systemic leaves. The amplification was done by RT-PCR with CMV-CP gene specific primers (Kumar et al. 2009). Lanes N = healthy plant as control, P = CMV-infected tobacco culture as positive control, 1–4 = leaf samples from four representative test plants exhibiting severe mosaic symptom. M = Lambda genome *EcoR*I/*Hind*III digested as DNA marker (Thermo Fisher Scientific India Pvt. Ltd., India). **Fig B: Production of H_2_O_2_ in CMV-infected *N*. *tabaccum* cv. White Burley plants.** A higher level of H_2_O_2_ (represented by brown color in leaf) was detected in CMV-infected tobacco plants followed by control, B-30488+CMV infected and B-30488 treated plant leaves. **Fig C: Polyphenol accumulation in different tissues of *N*. *tabaccum* cv. White Burley plants.** Anatomical features of tobacco stem (CS), (a) control (b) B-30488 (c) CMV-infected (d) B-30488+CMVinfected plants showing polyphenol accumulation on outer rows of cells and a casparian with thickened walls in virus infected plants.
(DOCX)
