# Supplementary material for: Paenibacillus lentimorbus Inoculation Enhances Tobacco Growth and Extenuates the Virulence of Cucumber mosaic virus
Source: PLoS One. 2016 Mar 2;11(3):e0149980. doi: 10.1371/journal.pone.0149980 (PMC4774868; doi:10.1371/journal.pone.0149980)
Supplement: S1 File — Table A: Details of primers used for this study. Table B: List of selected primer pairs of N. tabacum used for real time-PCR. Fig A: Detection of CMV in N. tabaccum cv. White Burley plants. Agarose gel image showing ~650 bp amplification from leaf samples of CMV-infected N. tabaccum cv. White Burley plants confirming the presence of virus in systemic leaves. The amplification was done by RT-PCR with CMV-CP gene specific primers (Kumar et al. 2009). Lanes N = healthy plant as control, P = CMV-infected tobacco culture as positive control, 1–4 = leaf samples from four representative test plants exhibiting severe mosaic symptom. M = Lambda genome EcoRI/HindIII digested as DNA marker (Thermo Fisher Scientific India Pvt. Ltd., India). Fig B: Production of H2O2 in CMV-infected N. tabaccum cv. White Burley plants. A higher level of H2O2 (represented by brown color in leaf) was detected in CMV-infected tobacco plants followed by control, B-30488+CMV infected and B-30488 treated plant leaves. Fig C: Polyphenol accumulation in different tissues of N. tabaccum cv. White Burley plants. Anatomical features of tobacco stem (CS), (a) control (b) B-30488 (c) CMV-infected (d) B-30488+CMVinfected plants showing polyphenol accumulation on outer rows of cells and a casparian with thickened walls in virus infected plants. (DOCX) [file pone.0149980.s001.docx]

**S1 File: Supplementary figures and tables**

***Paenibacillus lentimorbus* inoculation enhances tobacco growth and extenuates the virulence of *Cucumber mosaic virus***

Susheel Kumar^1#^, Puneet Singh Chauhan^2#^, Rashmi Raj^1^, Lalit Agrawal^2^, Ashish Srivastava^1^, Swati Gupta^2^, Shashank Kumar Mishra^3^, Sumit Yadav^2^, Poonam C. Singh^2^, Shri Krishna Raj^1^*, Chandra Shekhar Nautiyal^2^*

^1^ Plant Molecular Virology Laboratory, Council of Scientific and Industrial (CSIR)-National Botanical Research Institute (NBRI), Rana Pratap Marg, Lucknow-226 001 (UP), India

^2^ Division of Plant Microbe Interaction, CSIR-NBRI, Rana Pratap Marg, Lucknow-226 001 (UP), India

*Corresponding author’s E-mail:

SKR: skraj2@rediffmail.com;

CSN: nautiyalnbri@lycos.com;

Phone: +91-522-2205848; Fax: +91-522-2205839

^#^ These authors contributed equally to this work.

**Figure A. Detection of CMV in *N. tabaccum* cv. White Burley plants.** Agarose gel image showing ~650 bp amplification from leaf samples of CMV-infected *N. tabaccum* cv. White Burley plants confirming the presence of virus in systemic leaves. The amplification was done by RT-PCR with CMV-CP gene specific primers [26]. Lanes N = healthy plant as control, P = CMV-infected tobacco culture as positive control, 1-4 = leaf samples from four representative test plants exhibiting severe mosaic symptom. M = Lambda genome *EcoR*I/*Hind*III digested as DNA marker (Thermo Fisher Scientific India Pvt. Ltd., India).

**Figure B. Production of H_2_O_2_ in CMV-infected *N. tabaccum* cv. White Burley plants.** A higher level of H_2_O_2_ (represented by brown color in leaf) was detected in CMV-infected tobacco plants followed by control, B-30488+CMV infected and B-30488 treated plant leaves.

**Figure C. Polyphenol accumulation in different tissues of *N. tabaccum* cv. White Burley plants.** Anatomical features of tobacco stem (CS), (a) control (b) B-30488 (c) CMV-infected (d) B-30488+CMVinfected plants showing polyphenol accumulation on outer rows of cells and a casparian with thickened walls in virus infected plants.

**Table A.** Details of primers used for this study.

| **Primer** | | **Nucleotide sequence (5’-3’)** | **Annealing temperature (^o^C)** | **Product length**  **(bp)** |
| --- | --- | --- | --- | --- |
| CMV-CP | CMV-CP:F | GCATTCTAGATGGACAAATCTGAATC | 56 | 657 |
|  | CMV-CP:R | GCATGGTACCTCAAACTGGGAGCAC |  |  |
| EF1α | EF1α:F | TTGATATTGCGCTGTGGAAA | 58 | 428 |
|  | EF1α:R | GGTCGGGCCTTTATACCAAT |  |  |

F = Forward primer.

R = Reverse primer.

**Table B.** List of selected primer pairs of *N. tabacum* used for real time-PCR.

| **Gene** | **Primer name** | **Sequence (5’-3’)** |
| --- | --- | --- |
| Elongation factor 1-alpha (EF1a) | NtEF1a:F | TCGCCTTGTGGAAGTTTGAGAC |
|  | NtEF1a:R | CACCAACAGCAACAGTTTGACG |
| RNA-dependent RNA polymerase 2 (RdRP2) | NtRdRP2:F | CAGCGGGACAACAGGAGGTATTTT |
|  | NtRdRP2:R | AAGTAACATGATACCACGCCGATG |
| ZF-HD homeobox protein (ZF-HD) | NtZF-HD:F | AGATGCTCTAAAATGTGCTGCTTG |
|  | NtZF-HD:R | TCTCCTTTTGGTCTTGTGTGAACT |
| Asparagine synthetase (AsSyn) | NtAsSyn:F | GAGCGAGTGTGGCGTGTAGC |
|  | NtAsSyn:R | CCCATTAGCCATAGCAGGTTCA |
| Pathogenesis-related protein 1 (PR1) | NtPR1:F | TCTCAACAAGACTATTTGGATGCC |
|  | NtPR1:R | GCATAGGCTGCTACCTGGTCGTCC |
| Tetrahydrocannabinolic acid synthase (TCAS) | NtTCAS:F | TAGCACAGTGGAATGAAGAGGACG |
|  | NtTCAS:R | CAGCATCAACAGAGGAAGAAGGAC |
| Disease resistance protein (ADR1) | NtADR1:F | GGACTGGTTCAGAATGGATTGCCC |
|  | NtADR1:R | AGTCGGACAGGTGAGTGACGGATA |
| Pectin methylesterase inhibitor protein (PMI) | NtPMI:F | GGCTCGTGCTGCTTTATCAGTTAG |
|  | NtPMI:R | GCAGTCCTTTACGGCTTGTTTTTC |
| Brassinosteroid signaling kinase (BRSK) | NtBRSK:F | AAGGTGATGTAGTGTTTTTGGGGT |
|  | NtBRSK:R | CATCGCAAACTAAACTACTGAGGG |
| Copper/zinc superoxide dismutase (SOD) | NtSOD:F | GCAGATTCCTCTTGCTGGTC |
|  | NtSOD:R | CTTCCACCAGCATTTCCAGT |
| Catalase (CAT) | NtCAT:F | TAGTGCCAAAGGGTTTTTCG |
|  | NtCAT:R | ATCACGGATGAAGAAGACGG |
| Extracellular Beta-1, 3 glucanase gene (Gluc) | NtGluc:F | CAACACTGCTGATGTCCCAC |
|  | NtGluc:R | AGGCCAGCCACTTTCAGATA |

F = Forward primer.

R = Reverse primer.

Nt = *Nicotiana tabacum*.
